# *N* ^6^‐methyladenosine‐RNA methylation promotes expression of solute carrier family 7 member 11, an uptake transporter of cystine for lipid reactive oxygen species scavenger glutathione synthesis, leading to hepatoblastoma ferroptosis resistance

**DOI:** 10.1002/ctm2.889

**Published:** 2022-05-23

**Authors:** Ronald A. Hill, Yong‐Yu Liu

**Affiliations:** ^1^ School of Basic Pharmaceutical and Toxicological Sciences the University of Louisiana at Monroe Monroe Louisiana USA

**Keywords:** cystine, ferroptosis, hepatoblastoma, METTL3, RNA methylation, SLC7A11 transporter

## COMMENTARY

Hepatoblastoma (HB) is the most common liver tumour in children, accounting for approximately 66% of hepatic cancers in children and adolescent populations. The survival rate at 5 years of children afflicted with metastatic HB has steadily increased to currently 79%, but the prognosis remains poor for at least 20% of HB cases.^1^ Although HB is more responsive to chemotherapy than is hepatocellular carcinoma, failure of chemotherapy remains a prominent limitation in the management of HB patients.^2^

Overexpression of the antiporter solute carrier family 7 member 11 (SLC7A11) is prevalently extant in HB and associated with poor prognoses. The article entitled “*N* ^6^‐methyladenosine modification inhibits SLC7A11 mRNA deadenylation and enhances ferroptosis resistance in hepatoblastoma” by Liu et al., published in Clinical and Translational Medicine in May 2022, offers up convincing evidence that SLC7A11 overexpression attributes to enhanced *N* ^6^‐methyladenosine (m^6^A) presence in its mRNA transcripts. These findings further add crucial mechanistic enlightenment showing that m^6^A methylation catalyzed by methyltransferase‐like 3 (METTL3) causes overexpression of SLC7A11 to promote ferroptosis resistance. This work incepts translational discovery efforts while casting light on new prospective avenues for devising effective therapies to combat cancers.

**FIGURE 1:**
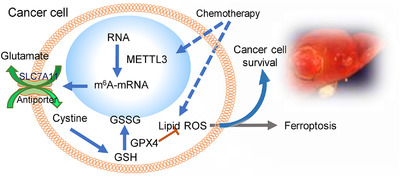
*N* ^6^‐methyladenosine (m6A) methylation upregulates expression of solute carrier family 7 member 11 (SLC7A11) and results in ferroptosis resistance. Chemotherapeutic agents can enhance m^6^A‐RNA methylation and cause metabolic stress with generation of reactive oxygen species (ROS) as well as phospholipid hydroperoxides (PLOOH) (lipid ROS) in cancer cells. Increased levels of lipid ROS can trigger (→) iron‐dependent ferroptosis to kill cancer cells; however, reduced glutathione (GSH) synthesized from intracellular cystine can constrain (┬) lipid ROS and avert ferroptotic death. Enhanced *N* ^6^‐methyladenosine (m^6^A) RNA methylation catalyzed by methyltransferase‐like 3 (METTL3) enhances the stability of SLC7A11 mRNA, thus upregulating its translation to protein, in turn increasing uptake of cystine for GSH synthesis

Cancer cells require more iron for rapid proliferation and survival in comparison to normal cells.^3^ Ferroptosis is an iron‐dependent form of cell death, distinct from apoptosis in that it can be initiated by metabolic stress. Once resistance to ferroptosis emerges, however, it remains unclear what metabolic determinants are critical for promoting such resistance or for enhancing tumorigenesis.^4^ The lethal trigger of ferroptosis is redox state imbalance with increased levels of intracellular reactive oxygen species (ROS), once cells are under stress, such as from chemotherapy.^5^ Increased ROS levels with a concomitantly elevated labile iron pool result in the peroxidation of membrane phospholipids to produce phospholipid hydroperoxides (PLOOH; lipid ROS), which then causes membrane instability and permeabilization, eventually leading to ferroptosis (sketched in Figure 1). Reduced glutathione (GSH) is one of the most important scavengers of ROS, and its elevated ratio *vs*. oxidized glutathione (GSSG) is a marker of oxidative stress. SLC7A11 (a.k.a. xCT) is a catalytic subunit of a cystine/glutamate antiporter that effects the uptake of extracellular cystine into cells in exchange for glutamate.^6^ Intracellular cystine can be rapidly reduced to cysteine for the synthesis of glutathione (GSH), which thereby serves as a co‐factor for glutathione peroxidase (GPX4) to reduce lipid peroxide, constraining PLOOH levels that would otherwise trigger ferroptotic death of cancer cells (Figure 1). In this way, SLC7A11, as an uptake carrier of cystine for lipid ROS scavenger (GSH) synthesis, plays a critical role in protecting cells from ferroptosis (Figure 1).^6^, ^7^ Besides HB, overexpression of SLC7A11 is often associated with resistance to chemotherapy and radiotherapy in other types of cancers, including Burkitt's lymphoma, and breast cancers.^4^, ^8^

This study, for the first time, offers substantive proof that overexpression of SLC7A11 in HB is crucial for tumorigenesis. SLC7A11 mRNA expression was pronounced in HB samples (*n* = 35 cases), as compared to matched normal liver; transcriptomics assays revealed SLC7A11 expression to be approximately 50‐fold higher in HB samples. Transfecting cancer cells (HuH6, HepG2) with the SLC7A11 gene significantly increased cell proliferation and colony formation, and these SLC7A11 knock‐in cells displayed resistance to erastin‐induced ferroptosis, attributable to decreased levels of lipid ROS. Conversely, silencing SLC7A11 expression with small interfering RNA (SLC7A11 siRNA) substantially decreased the expression of SLC7A11 in cells, significantly decreased cell proliferation and colony formation, and increased sensitivity to erastin‐induced ferroptosis. Further, silencing SLC7A11 expression with small hairpin RNA significantly decreased tumorigenesis of mice‐borne HuH6 xenotransplanted cells.

Elucidating a key molecular mechanism by which HB might be addicted to SLC7A11, an uptake antiporter of cystine for producing the lipid ROS scavenger GSH, holds great promise for the discovery of more efficacious and selective treatment approaches. RNA methylation (m^6^A modification) is mainly catalyzed by METTL3/METTL14 in human cancer cells.^9^ Aberrant m^6^A‐RNA can dysregulate RNA splicing, translocation, stability, and translation. Given that m^6^A occurs in approximately 25% of mRNA transcripts, and is prominently enriched in or around 5′‐ and 3′‐untranslated regions, and within long internal exons, the epigenetic impacts of locus‐specific m^6^A tags on cell character (such as tumorigenic or tumour‐suppressive) intrinsically accrue to the overall functions of the proteins encoded by the corresponding genes and their transcripts (noting here that a single gene may code for more than one protein product).^9^, ^10^ As evidenced by the results of Liu et al., m^6^A modification occurs in approximately 3.7% of the differentially expressed genes in human HB (153/4155 genes), based on mRNA sequence and MeRIP‐sequencing. Of the top 70 overlapping gene transcripts, SLC7A11 exhibited particularly high m^6^A‐RNA modification, prominently within 5′‐ and 3′‐untranslated regions. METTL3 in HB was markedly overexpressed, in parallel with SLC7A11 overexpression. In this study, elevated METTL3 expression was associated with promoted proliferation of HB cells, in accord with findings that pronounced METTL3 mRNA levels in HB tumours correlated with decreased survival rate among patients. Conversely, suppressing m^6^A levels via METTL3‐siRNA treatments suppressed SLC7A11 expression, allowing increased accrual of lipid ROS and consequent induced ferroptosis, thus preventing tumorigenesis of HB cancer cells and tumour growth in mice. Moreover, silencing METTL3 with siRNA enhanced the sensitivity of cancer cells to erastin treatments, and similarly for xenograft tumours in erastin‐treated mice. This evidence indicates that m^6^A methylation plays a critical role in promoting tumorigenesis of HB. The mechanistic studies further indicate that aberrant m^6^A presence can enhance SLC7A11 mRNA stability by inhibiting IGF2BP1 binding‐enabled degradative deadenylation, resulting in overexpression of SLC7A11 protein, thereby promoting ferroptosis resistance.

Discovery of therapeutic approaches for selectively killing cancer cells with emergent resistance is always a meritorious goal. Aberrant metabolic stress with oncogenes often presents in cancers, and constraining lipid ROS buildup provides cancer cells with a crucial enabler for evading ferroptosis in redox imbalance with supra‐normal iron uptake. Altogether the findings of Liu et al. strongly suggest that selective inhibition of m^6^A‐RNA methylation could offer an alternative means for targeting SLC7A11 overexpression for improving the treatments of HB or other cancers.
